# Identification of COVID-19–Associated Hepatitis in Children as an Emerging Complication in the Wake of SARS-CoV-2 Infections: Ambispective Observational Study

**DOI:** 10.2196/48629

**Published:** 2024-10-11

**Authors:** Sumit Kumar Rawat, Ajit Anand Asati, Nitu Mishra, Ashish Jain, Radha Kanta Ratho

**Affiliations:** 1 Bundelkhand Medical College and Hospital, Sagar, India; 2 Post Graduate Institute of Medical Education and Research, Chandigarh, India

**Keywords:** COVID-19, coronavirus, SARS-CoV-2, liver, hepatic, hepatitis, child, children, pediatric, pediatrics, retrospective, observational, jaundice, youth, inflammatory, inflammation

## Abstract

**Background:**

Although the pediatric population has largely remained free of severe COVID-19 symptoms, in some cases, SARS-CoV-2 infection has been associated with complications such as multiple inflammatory syndrome in children (MIS-C). We identified another a unique form of hepatitis occurring subsequent to asymptomatic SARS-CoV-2 infection, designated by us as COVID-19–associated hepatitis in children (CAH-C), in a subset of children who presented with hepatitis.

**Objective:**

Our study describes the clinical presentations, temporal association, and viral parameters of the CAH-C cases and compares them to those of MIS-C cases or other known forms of hepatitis in children.

**Methods:**

In an ambispective (retrospective and follow-up) observational study, records from April to July 2021 were reviewed for all children aged ≤14 years who were previously healthy and presented with a sudden onset of hepatitis, elevated transaminases, and nonobstructive jaundice. After performing all routine tests, those lacking marked inflammatory responses and without evidence of (1) other known causes of acute hepatitis or previous underlying liver disease and (2) multisystem involvement were classified as having CAH-C. Their characteristics were compared to those of children with MIS-C or other known forms of hepatitis.

**Results:**

Among the 5539 children tested for SARS-CoV-2, a total of 475 (8.6%) tested positive and 47 (0.8%) presented with hepatitis. Among the 47 children with hepatitis, 37 (79%) had features of CAH-C: having symptoms of hepatitis only, without protracted illness (mean length of stay 5 d), and an uneventful recovery following supportive treatment. In contrast, the remaining 10 (21%) had features of MIS-C–associated hepatitis: multiple system involvement; protracted illness (mean length of stay 8 d); and requiring admission to critical care, with a mortality rate of 30% (3/10).

**Conclusions:**

Our data suggest that CAH-C might be one of the new clinical complications associated with the emergence of newer variants of concern of SARS-CoV-2, which often result in changing presentations. Our findings should facilitate its early identification and thorough workup and aid its differentiation from other emerging syndromes in children, which would help initiate appropriate measures, enable better resource prioritization, and thus limit adversities.

## Introduction

SARS-CoV-2, the source of the COVID-19 pandemic, was first identified in China in December 2019. Its subsequent epicenter was recognized in the West, and thereafter, it reached India, causing the first wave of infections [1]. Interestingly, during the initial phase of the pandemic, children were disproportionately spared from severe illness with a predominance of asymptomatic or mild cases, obviating the need for hospitalizations [2,3]. Subsequently, multisystem inflammatory syndrome in children (MIS-C) was first observed in children and adolescents in the United Kingdom in April 2020, raising alarms about their safety, particularly in the absence of vaccines approved for children [4,5]. This was soon followed by detailed reports and linkage of MIS-C to SARS-CoV-2 [6]. The initial phase of pandemic caused numerous infections among children in India but no serious illness [7]. However, during the massive second wave of COVID-19 in April 2021, cases involving children with severe illnesses increased [8]. Newer variants of concern including the Delta (B.1.617.2) variant, which spread across the globe from India, were largely responsible for this massive upsurge [9]. This sometimes resulted in serious disease presentations varying from respiratory disease to entities such as MIS-C, encephalitis, and acute hepatitis in children [10,11].

Although initial reports seldom mention hepatitis as a one of the features or associated complications of COVID-19 [12], this finding was recognized in the later part of 2020. It has become crucial to delve into the nuances of hepatitis as a potential complication of COVID-19, especially considering the evolving landscape of our understanding about its interplay with host immunity and the environment. It has also been observed in a subset of children with obesity and MIS-C that severe disease and adverse outcomes were associated with coexisting hepatitis, similar to the findings observed among adults with COVID-19 [13]. However, about 60% of children with MIS-C presented with hepatitis compared to only 20% to 30% of adult cases admitted with COVID-19, which has been linked to severe disease course among such patients [14,15]. These findings indicate that age-related differences might exist in cases of hepatitis and the associated disease phenotypes. Moreover, its interplay with host immune response, the environment, and various other factors might have a bearing on the resulting morbidity, its management strategies, disease burden, and outcomes [16]. During active infection, a milder disease phenotype was observed without necessitating hospitalization, whereas receding infections were seen with more severe patterns such as MIS-C, often associated with a prolonged need for hospital admission.

During the ongoing phase of the pandemic, apart from children with MIS-C, there was another group of children presenting with hepatitis. This unique presentation of hepatitis was temporally associated with SARS-CoV-2 infections, which is designated by us as COVID-19–associated hepatitis in children (CAH-C). Such hepatitis cases outnumbered MIS-C cases and lacked the hallmarks of inflammation seen in MIS-C. CAH-C’s form of transient hepatitis is different from already familiar liver injury phenotypes and occasionally witnessed persistent diseases in children with COVID-19.

In the current context, cases of severe pediatric hepatitis of unknown origin have affected at least 169 children in nearly 12 countries, with at least 16 children having required liver transplantation [17]. The effects of liver injury are unexplained by either SARS-CoV-2 or adenovirus alone in the absence of common factors that cause hepatitis A and E. The children neither had a history of any toxic drug intake nor had exposure to common environmental pollutants causing hepatitis [18]. The underlying mechanisms; the role of a cofactor in play, if any; and the respective dominant hepatic phenotype in such cases may have a link with emerging variants of concern including the Delta, Omicron, or further variants [19]. Early identification of such entities is critical for early response, resource allocation, and formulation of guidelines for appropriate management. We hypothesized that CAH-C cases might have unique laboratory and clinical findings and outcomes compared to those of MIS-C cases. Hence, this study was planned with the aim to correctly identify such cases, find out its temporal relation with SARS-CoV-2 infection, describe its clinical and laboratory features, and differentiate it from the other similar entities.

## Methods

### Sample and Data

**Figure 1.**
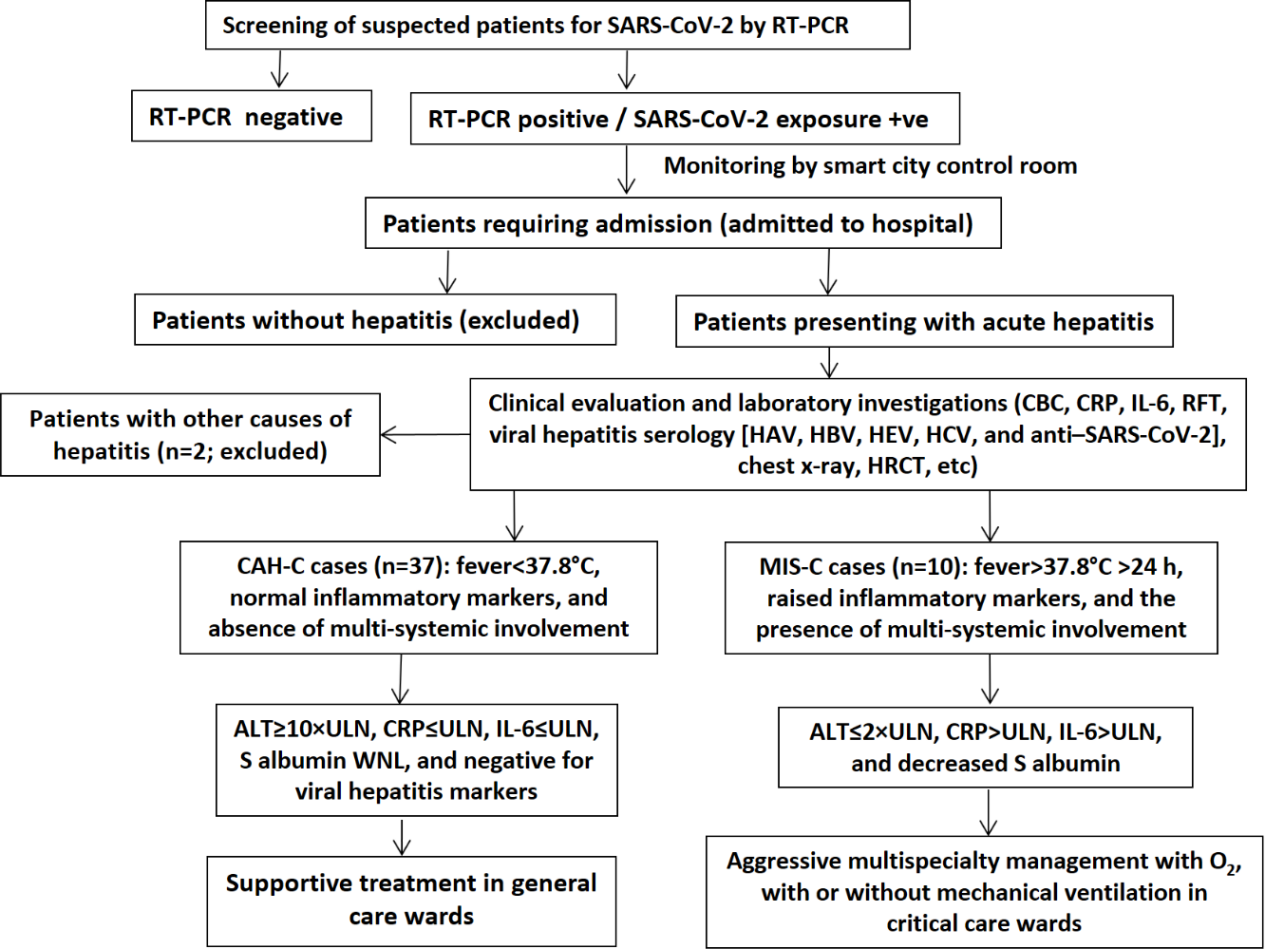
Study workflow and case workup. ALT: alanine aminotransferase; CAH-C: COVID-19–associated hepatitis in children; CBC: complete blood count; CRP: C-reactive protein; HAV: hepatitis A virus; HBV: hepatitis B virus; HCV: hepatitis C virus; HEV: hepatitis E virus; HRCT: high-resolution computed tomography; IL-6: interleukin-6; MIS-C: multiple inflammatory syndrome in children; O_2_: oxygen; RFT: renal function test; RT-PCR: reverse transcriptase–polymerase chain reaction; ULN: upper limit of normal; WNL: within normal limits.

In an ambispective (retrospective and follow-up) observational study, records from April to July 2021 were reviewed at a tertiary care public hospital in Central India, which was designated as a dedicated COVID-19 (500 beds) center for the entire region. Among persons who tested positive for COVID-19 by Indian Council of Medical Research (ICMR)–recommended reverse transcriptase (RT)–polymerase chain reaction (PCR) assays, children (≤14 years) who presented with serious symptoms including hepatitis and were admitted to the hospital were considered. The study reviewed the records of all such cases along with follow-up information collected after 4 weeks, and consent was taken as per the existing protocols as shown in Figure 1. These children were mainly referred through the central control command telemedicine center from the smart city control room of the district [20].

- Inclusion criteria: Children aged ≤14 years with laboratory evidence of COVID-19 during the study period were included.
- Exclusion criteria: Those with evidence of preexisting liver disease, drug-induced liver damage, or known cause of acute hepatitis were excluded.

### Measures

We analyzed all the data of these cases and separated those who presented with hepatitis during the study period. For these cases, SARS-CoV-2 RT-PCR screening tests were performed at the ICMR-recognized Virology Research and Diagnostic Laboratory of the hospital. All positive cases and those with suspected COVID-19 presenting with hepatitis were thoroughly evaluated as per the standard protocols, including complete blood counts; liver function tests; renal function tests; and tests for serum ferritin, C-reactive protein (CRP), D-dimer, lactate dehydrogenase, interleukin-6 (IL-6), myocardial enzymes, and procalcitonin at the central clinical laboratory of the hospital. Subsequent to this initial assessment, these children were subjected to a second round of SARS-CoV-2 RT-PCR testing.

Additionally, for the workup of hepatitis, the samples from these children were subjected to a battery of supplementary tests, including adenovirus PCR, human herpes simplex virus types 1 and 2 RT-PCR, hepatitis B surface antigen, anti–hepatitis A virus immunoglobulin (Ig) M, anti–hepatitis E virus IgM, anti–hepatitis C virus antibody, anti-*Leptospira* IgM, anti–Epstein Barr virus IgM, anti–varicella-zoster IgM, Widal test, malaria antigen, malaria antibody, antinuclear antibody, anti–liver kidney microsomal antibody, total IgG, anti–SARS-CoV-2 IgG, dengue NS1 antigen, and dengue IgM using ICMR-recommended enzyme-linked immunosorbent assay kits [21].

The analyzed cases were categorized into 2 distinct entities, each scrutinized on the basis of factors such as the temporal association with COVID-19, disease severity (including admission to the intensive care unit), levels of inflammatory markers, and the presence or absence of multisystem involvement, as determined by clinical, laboratory, and radiological findings.

1. CAH-C: Those aged ≤14 years; with laboratory evidence of recent COVID-19; presenting with a sudden onset of hepatitis, elevated transaminases, and nonobstructive jaundice; lacking marked inflammatory responses; and without evidence of (1) other known causes of acute hepatitis or previous underlying liver disease and (2) multisystem involvement
2. MIS-C–associated hepatitis: Cases of MIS-C within definitions according to the Centers for Disease Control and Prevention, presenting along with acute hepatitis, that is, elevated transaminases [22]

All patients were treated per the ICMR-recommended COVID-19 regimen for children: they were admitted to general or critical care wards, and other supportive treatment were provided as necessary.

The assessment of outcomes was based on the patients’ status at 4 weeks after discharge, serving as the end point. This timeframe was chosen in alignment with the routine follow-up protocol in place, which was guided by existing instructions from the state government to the district command center, ensuring consistency and adherence to established procedures.

### Data and Statistical Analysis

The data were extracted and entered into Microsoft Excel, and proportions and percentages were calculated for categorical variables. The *χ*^2^ test was performed to find possible associations between the categorical variables, such as the association of male sex and clinical outcome with either of the 2 entities. After checking the normality of data, the *t* test was applied to compare the mean values of different parameters. For variables without normality, the Mann-Whitney *U* test or Fischer exact test was applied. A *P* value of less than .05 was considered significant. The statistical analysis was performed using SPSS software (trial version 16 for Windows; IBM Corp).

### Ethical Considerations

The follow-up and analysis work was performed after obtaining the approval of the human ethics committee of Bundelkhand Medical College, Sagar (reference IEC/BMC/80/21). A consent waiver was granted for the use of anonymized data. The provision of compensation was not required as our study was observational in design.

## Results

### Patient Characteristics

During the study period (from April to July 2021), among the 5539 screened children, where the youngest child was 2 months old and the oldest children were 14 years old, with a mean age of 9 (SD 3.8) years, 2984 (53.9%) were male and 2555 (46.1%) were female. Among them, 475 (8.6%) children were found to be positive for COVID-19 by RT-PCR, wherein the youngest child was 4 months old and the oldest children were 14 years old, with a mean age of 9 (SD 3.6 years); there were 303 (63.8%) male children and 172 (36.2%) female children (ratio 1.76:1).

A total of 86 children were admitted for various reasons to the hospital. Among them, 47 (56%) children who presented with features of hepatitis satisfied the inclusion criteria; 2 other children presenting with hepatitis were excluded from the study, as 1 of them was suspected to have drug-induced liver injury and the other was positive for hepatitis A virus infection (Figure 1). As per the abovementioned criteria, 37 patients (male: n=23, 64%; male:female ratio=1.64:1) were categorized as having CAH-C, and 10 patients (male: n=3, 30%; male:female ratio=0.42:1) were categorized as having MIS-C (Table 1).

The age of children who were admitted with presentation of hepatitis ranged from 4 months to 14 years, with a mean age of 6.6 (SD 3) years. The majority (20/47, 43%) of these children belonged to the 6‐11 years age group. The age distribution of children with CAH-C and those with MIS-C is shown in Table 2. There were no differences in their ethnicity or racial characteristics, and none of these children had any travel history.

**Table 1.** Comparison of patient characteristics of CAH-C^a^ versus MIS-C^b^-associated hepatitis cases from April to June 2021.

Patient characteristics		CAH-C cases (n=37)	MIS-C cases (n=10)	*P* value
Age (years), mean (SD)		6.75 (3.32)	6.2 (4.29)	.34^c^
Male sex, n (%)		23 (64)	3 (30)	.08^d^
Total number of days admitted, mean (SD)		4.64 (1.37)	8.4 (1.95)	<.001^c^
Days from SARS-CoV-2 exposure, mean (SD)		23.56 (6.63)	9.7 (93.74)	<.001^c^
**Clinical outcome, n (%)**				
	Recovered	37 (100)	7 (70)	.01^d^
	Death	0 (0)	3 (30)	.01^d^

a CAH-C: COVID-19–associated hepatitis in children*.*

b MIS-C: multisystem inflammatory syndrome in children.

c Independent samples *t* test.

d Fischer exact test.

**Table 2.** Age distribution of children with CAH-C^a^ versus those with MIS-C^b^.

Age group (years)	CAH-C cases (n=37), n (%)	MIS-C cases (n=10), n (%)
Infants (0‐1)	0 (0)	1 (10)
Toddlers (1‐3)	7 (19)	3 (30)
Preschoolers (4‐5)	7 (19)	1 (10)
Middle schoolers (6‐11)	17 (46)	3 (30)
Adolescents (12‐14)	6 (16)	2 (20)

a CAH-C: COVID-19–associated hepatitis in children*.*

b MIS-C: multisystem inflammatory syndrome in children.

### Temporal Association

**Figure 2.**
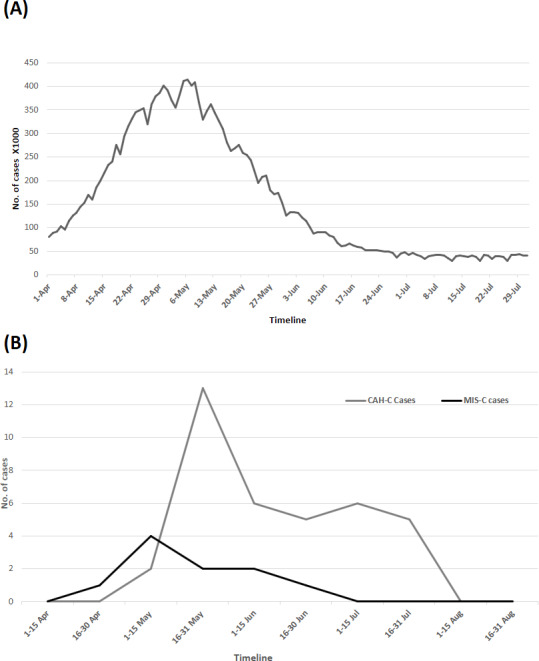
Temporal relationship between (A) new COVID-19 infections and (B) cases of hepatitis in children. Data source: Ministry of Health and Family Welfare, Government of India [23]. CAH-C: COVID-19–associated hepatitis in children; MIS-C: multisystem inflammatory syndrome in children.

Cases of CAH-C started appearing at the end of April and peaked at the end of May; they then showed a decline in trend, with a small number of cases still present 4 weeks after the disappearance of new SARS-CoV-2 infections in the district, and lagged behind MIS-C cases. Their temporal relationship to the incidences of SARS-CoV-2 infections in the district is shown Figure 2.

### Clinical Presentation and Laboratory Findings

**Figure 3.**
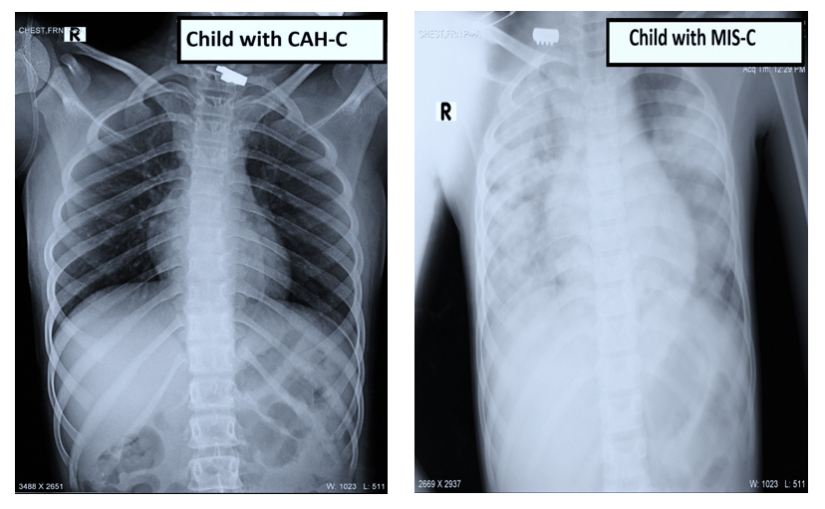
The x-ray findings of a child with CAH-C versus a child with MIS-C. CAH-C: COVID-19–associated hepatitis in children; MIS-C: multisystem inflammatory syndrome in children.

CAH-C cases presented with typical symptoms of hepatitis including nausea, vomiting, loss of appetite, weakness, and mild fever not exceeding 38°C. Two cases had signs of acute liver failure including altered sensorium. Ultrasound findings, wherever available (5/37, 14%), were suggestive of periportal and cholecystic involvement, with gallbladder wall thickening in 1 case. None of them had any significant finding on chest x-rays (Figure 3). MIS-C cases (n=10) with hepatitis presented with moderate to severe symptoms and a persistent fever of >38°C that caused them to be hospitalized. One (10%) child presented with epidermal necrosis and toxic shock–like imaging, 1 (10%) with encephalitis, and 5 (50%) with pneumonia on chest x-rays (Figure 3). Besides persistent fever, they had subconjunctival hemorrhages, cough, and shortness of breath, with features of hepatitis such as abdominal pain, loss of appetite, and weakness. Of them, 3 (30%) developed acute respiratory distress syndrome and signs of multiorgan failure, mainly hypotension—these cases all resulted in death; 1 (10%) developed encephalitis; and 1 (10%) developed signs of acute liver failure. All were admitted to intensive care units.

Regarding the laboratory workup, 95% (35/37) of CAH-C cases had a negative RT-PCR test for SARS-CoV-2 at the time of admission. The majority of cases (29/37, 78%) had substantially elevated transaminases (>10× the upper limit of normal; median 1326.2, range 70‐5685 U/L) and serum bilirubin (median 4.05, range 1.4‐17.1 mg/dL). In all, 68% (25/37) had unelevated CRP, whereas the remaining 32% (12/37) had moderate elevation (median 5.4, range 0.70‐7.9 mg/L). The median IL-6 value was 9.7 (range 2.1‐24.6.3) pg/mL (*P*<.001). Many (16/37, 43%) had elevated alkaline phosphatases 2× the upper limit of normal (*P*=.29), with slightly elevated total IgG levels (median 1245.3, range 551.4‐1640 mg/dL). A total of 95% (35/37) had normal platelet counts (median 2.45, range 0.69‐7.7 per mm^3^×10^3^). The 2 children who had thrombocytopenia also had signs of acute liver failure, elevated prothrombin time, and a raised international normalized ratio and D-dimer level. The international normalized ratio test could be performed in 65% (24/37) of cases, and the D-dimer test could be performed in 22% (8/37) of cases, among whom 3 were raised and 5 were within range (Table 3). All 37 (100%) patients were positive for SARS-CoV-2, with anti-N protein antibodies in high titers. The results for tests performed for pathogens are shown in Table 4 (also see Multimedia Appendix 1).

**Table 3.** Laboratory findings of CAH-C^a^ versus MIS-C^b^–associated hepatitis cases.

Laboratory values	CAH-C (n=37), median (IQR)	MIS-C (n=10), median (IQR)	*P* value^c^
CRP^d^ (mg/L)	4.10 (0.70-7.90)	17.85 (10.82-28.42)	<.001
IL-6^e^ (pg/mL)	9.70 (4.27-13.42)	134.40 (34.00-227.52)	<.001
Platelet count (per mm^3^×10^3^)	2.45 (2.10-3.06)	2.85 (1.35-3.85)	.89
TBil^f^ (mg/dL)	5.4 (2.95-7.15)	0.90 (0.87-1.37)	<.001
Albumin (g/dL)	3.60 (3.42, 3.67)	3.45 (2.70-3.77)	.39
AST^g^ (U/L)	942.45 (301.67-2002.05)	85.50 (47.55-127.90)	<.001
ALT^h^ (U/L)	1326.25 (492.12-2124.92)	76.00 (44.42-170.45)	<.001
Alkaline PO4 (U/L)	311.00 (187.40-498.20)^i^	200.75 (131.12-312.85)	.10
INR^j^	1.30 (1.05-1.52)^k^	1.00 (1.00-1.39)	.17

a CAH-C: COVID-19–associated hepatitis in children*.*

b MIS-C: multisystem inflammatory syndrome in children.

c Mann-Whitney *U* test.

d CRP: C-reactive protein.

e IL-6: interleukin-6.

f TBil: total bilirubin.

g AST: aspartate transaminase.

h ALT: alanine aminotransferase.

i n=27.

j INR: international normalized ratio.

k n=24.

**Table 4.** Results of the comprehensive pathogen testing performed in both groups of patients.

Tests performed	Positivity detected, n/N (%)	
	CAH-C^a^ cases (n=37)	MIS-C^b^ cases (n=10)
Anti–SARS-CoV-2 IgG^c^ (anti-N)	37/37 (100)	3/5 (60)
ANA^d^	0/37 (0)	1/5 (20)
Anti-LKM^e^	0/37 (0)	0/5 (0)
Anti-HAV^f^ IgM	0/37 (0)	1/8 (12)
HbSAg^g^	0/37 (0)	0/8 (0)
Anti-HCV^h^	0/37 (0)	0/8 (0)
Anti-HEV^i^ IgM	0/37 (0)	0/8 (0)
Anti-*Leptospira* IgM	0/37 (0)	0/5 (0)
Anti-VZV^j^ IgM	7/37 (19)	0/5 (0)
Anti-EBV^k^ IgM	8/37 (22)	0/5 (0)
*Salmonella sp.* (Widal titer >160)	21/37 (57)	0/8 (0)
Dengue IgM	8/37 (22)	0/5 (0)
HSV^l^ 1 and 2 (PCR^m^)	0/17 (0)	0/3 (0)
Adenovirus (PCR)	3/17 (18)	0/3 (0)
SARS-CoV-2 (PCR)	34/34 (100)	7/10 (70)
SARS-CoV-2 (repeat or on admission)	2/37 (1)	3/8 (38)

a CAH-C: COVID-19–associated hepatitis in children*.*

b MIS-C: multisystem inflammatory syndrome in children.

c Ig: immunoglobulin.

d ANA: antinuclear antibody.

e LKM: liver kidney microsomal.

f HAV: hepatitis A virus.

g HbSAg: hepatitis B surface antigen.

h HCV: hepatitis C virus.

i HEV: hepatitis E virus.

j VZV: varicella-zoster.

k EBV: Epstein-Barr virus.

l HSV: herpes simplex virus.

m PCR: polymerase chain reaction.

As shown in results of the laboratory investigation in Table 3, for patients with MIS-C who presented with hepatitis, the majority (7/10, 70%) had a positive RT-PCR test for SARS-CoV-2 within 2‐3 weeks. They had markedly raised CRP (median 17.8, range 3.8-41.2 mg/L; *P*<.001), significantly elevated IL-6 (median 134.4, range 11.9-311.2 pg/mL; *P<*.001), and reduced albumin levels (median 3.3, range 2.4‐4.0 g/dL; *P*=.12). D-dimer level was elevated (>1.5) in all 7 cases in which it could be measured. They had reduced platelet counts in some cases, moderately elevated transaminases (alanine aminotransferase 40‐200 U/L), normal or borderline raised serum bilirubin, mildly elevated alkaline phosphatases, and elevated procalcitonin (available for 4/10, 40% cases). Anti–SARS-CoV-2 IgG antibody test was positive in 60% (3/5) of cases; these 3 cases had a contact history with confirmed COVID-19 cases but were themselves RT-PCR negative.

### Treatment

CAH-C cases (n=37) who were admitted in noncritical care wards were given supportive therapy, consisting of antiemetics, intravenous fluids, vitamins, and zinc, without any requirement of oxygen administration or steroids. Those with MIS-C and hepatitis (n=10) were treated as per the ICMR-recommended COVID-19 regimen for children: admission to critical care and other supportive treatment, inclusive of intravenous Ig in a child with neurologic symptoms (n=1); steroids for all (n=10); and oxygen administration (n=3), with mechanical ventilation in 1 case.

### Outcomes

All patients admitted with CAH-C were discharged with supportive treatment and without any major complications, with a mean hospital stay of 4 (SD 1.0) days, and remained uneventful at the 4-week follow-up. No mortality was reported in this group. Among the 10 children classified as having MIS-C, 1 (10%) child developed paralytic ileus, which resolved on conservative treatment. However 3 (30%) children had an adverse outcome; 2 developed signs of acute respiratory distress syndrome and multiorgan failure, where 1 of them also showed signs of acute liver failure, and 1 developed encephalitis. The mean hospital stay in this group was 8 (SD 1) days. At the 4-week postdischarge follow-up, the remaining 7 (70%) children had recovered.

## Discussion

In the wake of recurrent waves of infections driven by newer variants of SARS-CoV-2, the pediatric population faces safety concerns owing to diverse symptoms and post–COVID-19 complications. In this context, our study identified 37 unique cases presenting with a distinctive manifestation, labeled as CAH-C, whereas MIS-C accounted for hepatitis in 10 cases among the 5539 children screened in the district during the study period. In this regard, the salient findings of the study are shown in Table 5.

**Table 5.** Significant differences between CAH-C^a^ and MIS-C^b^ cases from April to June 2021.

Patient characteristics among children with hepatitis	CAH-C cases (n=37)	MIS-C cases (n=10)
Total number of days of admission (protracted illness)	Fewer	More
Days from SARS-CoV-2 exposure	More (mean 23 days)	More (mean 8 days)
Outcomes	Better	Poor
Inflammatory markers (CRP^c^ and IL-6)^d^	Usually normal	Raised
Serum bilirubin	Markedly elevated	Usually normal
ALT^e^ and AST^f^	Markedly elevated	Slightly elevated

a CAH-C: COVID-19–associated hepatitis in children*.*

b MIS-C: multisystem inflammatory syndrome in children.

c CRP: C-reactive protein.

d IL-6: interleukin-6.

e ALT: alanine aminotransferase.

f AST: aspartate transaminase.

Cases of CAH-C were more frequently reported in older children (6‐11 y) and had male preponderance, in comparison to MIS-C cases, where comparatively younger and female children were more affected, although this predilection for female children in MIS-C versus the predilection for male children in CAH-C was not found to be statistically significant (*P*=.07).

This higher occurrence of CAH-C in male children may be due to higher expression of receptors, namely angiotensin-converting enzyme 2 in cholangiocytes [24], and transmembrane serine protease 2 in hepatocytes among male children might be the predisposing factor, warranting further evaluation [25]. It is important to note that this study is an initial report, and all cases involved individuals of South Asian ethnicity. Consequently, any conclusions regarding racial predisposition to this condition cannot be drawn until data from other regions of the world have been collected, reported, and compared.

With regard to temporal associations, the CAH-C cases manifested abruptly, emerging 2‐6 weeks after exposure to a laboratory-confirmed COVID-19 case, following an initial phase of asymptomatic or a mild symptomatic presentation, whereas patients with MIS-C had a gradual onset and emerged at around 2‐3 weeks after exposure, which is slightly earlier in comparison.

The CAH-C cases did not display markedly elevated inflammatory markers or systemic derangements during laboratory testing [26]. CAH-C cases exhibited raised anti–SARS-CoV-2 antibodies, significant Widal titers, positive dengue IgM, positive anti–Epsiten Barr virus IgM, and positive anti–varicella-zoster IgM. The multipositivity for various infectious agents, coupled with elevated total IgG levels, are unusual for children of such young age. These findings point to skewed immune activation, especially B cell stimulation giving rise to multiple antibody responses as a result of possible polyclonal B cell immune activation, likely initiated by some of the SARS-CoV-2 antigens or their possible cross-reactions with other infective agents. This possible role played by SARS-CoV-2 in CAH-C is in contrast to the polyclonal (Vβ 21.3+ CD4+ and CD8+) T cell stimulation observed in cases of MIS-C [27]. MIS-C is reported to be associated with the depletion of B cell responses along with polyclonal T cell responses; in contrast, we observed that CAH-C being on the milder spectra of disease course showed evidence of polyclonal B cell activation [28].

Currently, the antibodies seen in children with CAH-C seem to be more of a negative than positive effect that offers any protection, since children exhibit a narrow spectrum of reactivity against SARS-CoV-2, which may not necessarily be of a neutralizing nature. A similar situation has been reported to cause the persistence of symptoms in children and adults who recovered from COVID-19 due to alterations and persistence in B cell–mediated responses during convalescence [29].

Besides the raised inflammatory markers in MIS-C cases, this serological multipositivity for various infectious agents along with elevated total IgG levels were missing in children with MIS-C.

With regard to the outcomes among the 2 entities, the CAH-C cases were monitored with vigilance due to the absence of existing treatment guidelines, and they responded well to supportive care without a specific recommended COVID-19 treatment [30]. The cases exhibited a shorter hospital stay, fair recovery, and no mortality at the decided follow-up time point. In contrast, MIS-C cases, representing a more severe form, had higher mortality, a longer hospital stay, and worse outcomes as evidenced by the follow-up results, underscoring the critical nature of MIS-C [6].

However, this may represent just the tip of the iceberg, as our report is preliminary and primarily focused on prominent symptoms, potentially missing less conspicuous features. The anticipated further phases of infections in postpandemic times caused by the spread of further emerging variants of concern necessitate heightened vigilance.

Our study does have few limitations. We should mention that despite the apparent milder course of CAH-C, it would be premature to categorize it definitively as a less severe complication, especially considering the likelihood of biological false positivity, as reported in some other disease conditions often known to be caused by polyclonal B cell activation [31]. This consideration becomes critical in regions where febrile illnesses, including dengue, chikungunya, and enteric fever, remain endemic. A longer follow-up and experimental studies are essential to develop comprehensive treatment guidelines for this unique manifestation. Further extensive studies exploring epidemiology among larger cohorts in different geographical regions and detailed studies exploring its immunopathology are warranted.

In conclusion, the evolving landscape of SARS-CoV-2 variants introduces novel manifestations across age groups. Our research emphasizes the importance of timely recognition of cases presenting with hepatitis. New entities such as CAH-C should be identified early to enable physicians to prioritize cases, initiate specific therapies, prevent adverse outcomes, and reduce the strain on health care resources during the ongoing postpandemic transmission of the virus.

## Supplementary material

10.2196/48629 Multimedia Appendix 1 Baseline data at presentation for hepatitis cases in children.

## Abbreviations

CAH-C: COVID-19–associated hepatitis in children
CRP: C-reactive protein
ICMR: Indian Council of Medical Research
Ig: immunoglobulin
IL-6: interleukin-6
MIS-C: multiple inflammatory syndrome in children
PCR: polymerase chain reaction
RT: reverse transcriptase
